# Nucleolar stress regulation of endometrial receptivity in mouse models and human cell lines

**DOI:** 10.1038/s41419-019-2071-6

**Published:** 2019-11-04

**Authors:** Wei Hu, Yu-Xiang Liang, Jia-Mei Luo, Xiao-Wei Gu, Zi-Cong Chen, Tao Fu, Yu-Yuan Zhu, Shuai Lin, Hong-Lu Diao, Bo Jia, Zeng-Ming Yang

**Affiliations:** 1 College of Veterinary Medicine, South China Agricultural University, 510642 Guangzhou, China; 2 Laboratory Animal Center, Shanxi Key Laboratory of Experimental Animal Science and Animal Model of Human Disease, Shanxi Medical University, 030001 Taiyuan, China; 3 Shanxi Key Laboratory of Birth Defect and Cell Regeneration, Shanxi Medical University, 030001 Taiyuan, China; 4 Reproductive Medicine Center, Renmin Hospital, Hubei University of Medicine, 442000 Shiyan, China; 5 Jiangxi Provincial Institute of Occupational Medicine, 330006 Nanchang, China

**Keywords:** Infertility, Endocrine reproductive disorders

## Abstract

Embryo implantation is essential to the successful establishment of pregnancy. A previous study has demonstrated that actinomycin D (ActD) could initiate the activation of mouse delayed implantation. However, the mechanism underlying this activation remains to be elucidated. A low dose of ActD is an inducer of nucleolar stress. This study was to examine whether nucleolar stress is involved in embryo implantation. We showed that nucleolar stress occurred when delayed implantation was activated by ActD in mice. ActD treatment also stimulated the Lif-STAT3 pathway. During early pregnancy, nucleolar stress was detected in the luminal epithelial cells during the receptive phase. Blastocyst-derived lactate could induce nucleolar stress in cultured luminal epithelial cells. The inhibition of nucleophosmin1 (NPM1), which was a marker of nucleolar stress, compromised uterine receptivity and decreased the implantation rates in pregnant mice. To translate these mouse data into humans, we examined nucleolar stress in human endometrium. Our data demonstrated that ActD-induced nucleolar stress had positive effects on the embryo attachment by upregulating IL32 expression in non-receptive epithelial cells rather than receptive epithelial cells. Our data should be the first to demonstrate that nucleolar stress is present during early pregnancy and is able to induce embryo implantation in both mice and humans.

## Introduction

The cross-talk between the blastocyst with implantation competency and the receptive endometrium is critical for successful implantation^1–3^. Embryo implantation is a rate-limiting step for successful pregnancy. In humans, ~15% of couples are infertile. The failure of embryo implantation is the main cause of early pregnancy loss^1^.

Nucleolus, a distinct membrane-less subnuclear compartment, contains three morphologically distinct sub-compartments: the fibrillar center, the dense fibrillar component, and the peripheral granular component^4^. Nucleolus is primarily associated with ribosome biogenesis, the regulation of mitosis, cell-cycle progression, proliferation, and stress responses^5^. Nucleolar stress is the failure of ribosome biogenesis or the failure of a function that ultimately leads to a disturbed homeostasis^4^. Nucleolar stress can be used to monitor the synthesis and assembly of rRNAs and ribosome proteins, and can be induced by ActD, 5-FU, CX5461, nutrient deprivation, or ultraviolet radiation^6,7^. NPM1 is localized in the nucleolus under normal conditions^8–11^. Once nucleolar stress occurs, NPM1 relocates from the nucleolus to the nucleoplasm, where it can combine with Mdm2 (known as HDM2 in human) to prevent p53 from the ubiquitination degradation by Mdm2 and cause the accumulation of p53^8,11^. The roles of nucleolar stress in cancer cells have attracted significant attention^7,12^. However, the potential physiological functions of nucleolar stress remain largely unexplored.

The establishment of uterine receptivity is a key step for successful pregnancy^1,2^. Both LIF and Stat3 are essential to mouse blastocyst implantation^13–15^. p53 also plays an important role during embryo implantation via regulating LIF^16^. Under nucleolar stress, p53 accumulation is significantly enhanced^17^. It has been shown that ActD treatment is able to initiate the activation of delayed implantation in mice and rats^18,19^. Otherwise, embryo implantation process is accompanied by increased endometrial vascular permeability, cell damage and inflammatory response including IL-6, IL-1, and LIF^20^. Therefore, it is reasonable that there may exist some cell stress responses which may be essential during embryo implantation process. We hypothesized that nucleolar stress should play a key role during embryo implantation.

Our results show that nucleolar stress occurs during the receptive phase of early pregnancy and that the Lif-STAT3 signaling pathway is activated by nucleolar stress that is induced by blastocyst-derived lactate. NPM1 inhibition compromises mouse embryo implantation. Our data also showed that nucleolar stress in human non-receptive epithelial, but not receptive epithelial, could improve the attachment rates of Jeg-3 spheroids through increasing IL32 secretion.

## Materials and methods

### Animals and treatments

Mature CD1 mice were obtained from Hunan Slack Laboratory Animal Co., LTD and were maintained under a specific pathogen-free (SPF) and controlled environment (cycles of 12 h of light and 12 h of dark). All animal procedures were approved by the Animal Care and Use Committee of South China Agricultural University. Pregnant or pseudopregnant female mice (8–10 weeks) were obtained by mating with fertile or vasectomized males of the same strain (day 1 is the day of the vaginal plug). From days 1 through 4, the embryos were flushed from the oviducts or uteri to determine whether the mice were successfully pregnant. The implantation sites on day 5 were confirmed by a tail intravenous injection of Chicago blue dye (Sigma-Aldrich, USA). Artificial decidualization was performed as previously described^21,22^.

On day 4 (0800 h to 0900 h) of pregnancy, the mice were ovariectomized to induce delayed implantation. Delayed implantation was maintained by the daily injection of progesterone (1 mg/0.1 ml sesame oil/mouse, Sigma-Aldrich) from days 5 to 9. On day 7, the mice were intraperitoneally injected with ActD (27.5 μg/mouse (27.5 μg ActD dissolved in 2.191 μl DMSO and 97.809 μl saline), Abcam, UK). The injection of an equal solvent served as a control. The mice were killed at different time points after the injection to collect the uteri for further analyses.

To examine the effects of NPM1 on embryonic implantation, the mice were intraperitoneally injected with the NPM1-specific antagonist NSC348884 (80 μg/mouse and 160 μg/mouse in saline; 31.85 mg/ml dissolved in DMSO and diluted in saline, 100 μl per mouse, Selleckchem, USA) from days 4 to 5 of pregnancy. The injection of an equal solvent served as a control. On day 6, the implantation sites were confirmed by a tail intravenous injection of Chicago blue dye. To analyze the effects of NPM1 on the implantation-related markers, NSC348884 was injected on day 3 of pregnancy. The uteri were collected 24 h after NSC348884 injection.

### Isolation and treatment of uterine luminal epithelial cells

The luminal epithelial cells were isolated as previously described^23^. Briefly, to obtain the luminal epithelial cells, the uteri were digested with sterile HBSS containing 0.3% trypsin and 6 mg/ml dispase for 1.5 h at 4 °C followed by 30 min at room temperature and 10 min at 37 °C. The luminal epithelial cells were cultured in DMEM/F-12 medium (Sigma-Aldrich) containing 10% heat-inactivated fetal bovine serum (FBS, Invitrogen, USA). For in vitro treatment, the luminal epithelial cells were treated with associated reagents.

### Human cell lines and in vitro embryo attachment assay

Ishikawa (Human endometrial epithelial cells), Jeg-3 (HTB-36), and AN3 CA (Human endometrial epithelial cells) cells were purchased from American Type Culture Collection (ATCC, USA). The embryo attachment in vitro model was constructed as described previously with minor modifications^24^. Briefly, Ishikawa and Jeg-3 were cultured in DMEM/F-12 medium containing 10% FBS. AN3 CA were cultured in MEM (Eagle) medium (Sigma-Aldrich) supplemented with 10% FBS and L-Glu. The Ishikawa cells or AN3 CA cells were seeded at 2 × 10^5^ cells per well in 12-well plate. The Jeg-3 cells at about 90% confluency were trypsinized, washed and seeded (2.5 × 10^5^ cells per well) in a 6-well plate and shaken at 84 rpm overnight for spheroid generation. After treatment by ActD 10 nM or recombinant human IL32 (Bio Vision, USA) 10 ng/mL and 100 ng/mL for 48 h, the wells containing the Ishikawa cells were washed with PBS and refilled with fresh DMEM/F-12 containing 1% bovine serum albumin (BSA, Sigma-Aldrich). Jeg-3 spheroids with diameter of 100–200 μm were selected and 30 spheroids were gently added onto Ishikawa monolayer with a glass pipette under microscopic visualization per well. The co-culture was maintained for 1 h at 37 °C under 5% CO_2_ in air and then subjected to vigorous shaking at 140 rpm for 10 min. The medium was removed and refilled. The number of attached spheroids was counted under microscope. Attachment rate was defined as the ratio of the number of attached spheroids to the total number of spheroids seeded.

### Human endometrium sample collection

For experiments examining expression patterns of NPM1, we used samples of human endometrium from health women between the age of 18 and 45 with regular cycles. All human procedures were approved by the Institutional Committee on the Use of Human Subjects in Hubei University of Medicine. Written informed consent was obtained from all participating subjects that donated endometrial biopsies. Histologic dating of endometrial samples was done on the basis of the criteria of Noyes, and confirmed by subsequent histopathological examination.

### Transfer of ActD-soaked beads

The injections of ActD-soaked beads were performed as previously described^25,26^. Briefly, Affi-Gel Blue Gel Beads (#1537301, Bio-Rad, USA), which were approximately the size of a blastocyst, were washed three times with sterile Hanks’ balanced salt solution (HBSS, Sigma-Aldrich) and were then incubated with ActD (1 mM) in 30 μl 0.4% PVA (Sigma-Aldrich) at 37 °C for 3 h. Loaded beads (eight beads/ horn) were transferred into the uterine lumen of day 4 pseudopregnant mice. The mice were killed at day 6 after Chicago blue dye injection. The uteri with blue bands were collected for further analyses.

### In situ hybridization

The cDNA fragment of each target gene was amplified with specific PCR primers (Table 1) and was cloned into pGEM-T easy vector (Promega, USA). After ensuring the direction of each insert in the vectors, the antisense or sense cRNA probes were labeled with the Digoxigenin RNA Labeling Kit (Roche Applied Science, USA).

**Table 1:** Primers used in this study

Gene	Primers (5′-3′)	ID	Products (bp)	Application
*Rpl7*	GCAGATGTACCGCACTGAGATTC ACCTTTGGGCTTACTCCATTGATA	NM_29016	129	RT-PCR
*Its1*	TCCGTGTCTACGAGGGGCGG GGGTGCCGGGAGAGCAAAGC	XR_877120.2	95	RT-PCR
*p21*	TGAGCGGCCTGAAGATTCC TCTGC	NM_007669.5	82	RT-PCR
*Mdm2*	AATTTAGTGGCTGTAAGTCAGCAAGA ATCCTTCAGATCACTCCCACCTT	NM_010786.4	87	RT-PCR
*Lif*	AAAAGCTATGTGCGCCTAACA GTATGCGACCATCCGATACAG	NM_008501	98	RT-PCR
*p53*	GCAGTTGTGGGTCAGC ATCACCATCGGAGCAG	NM_011640	130	RT-PCR
*RPL7*	CTGCTGTGCCAGAAACCCTT TCTTGCCATCCTCGCCAT	NM_000971	194	RT-PCR
*IL32*	GACTTCAAAGAGGGCTACC GGCACCGTAATCCATCTC	NM_001012631.1	103	RT-PCR
*ITS1*	TGTCAGGCGTTCTCGTCTC GAGAGCACGACGTCACCAC	NR_046235.1	146	RT-PCR
*Npm1*	AGCACCAGTTGTCATTAAGA CTCATCATCGTCCTCATCAT	NM_001252260.1	386	ISH
*Ptgs2*	TGGCTTCGGGAGCACAAC GCCTTTGCCACTGCTTGT	NM_011198	437	ISH
*Wnt4*	GGAGACGTGCGAGAAACTCA TGTTGTCCGAGCATCCTGAC	NM_009523.2	356	ISH
*Egr1*	CCCATGATCCCTGACTATCT CAAACTTCCTCCCACAAAT	NM_007913	469	ISH

In situ hybridization was performed as described previously^25,27^. Frozen sections (10 μm) were fixed with a 4% paraformaldehyde solution in PBS and were hybridized overnight at 55 °C. Following the post-hybridization washes, the sections were incubated with a sheep anti-digoxigenin antibody conjugated to alkaline phosphatase at 4 °C overnight (1:5000; Roche). Finally, the signals were visualized with nitroblue tetrazolium (NBT, 0.4 mM) and 5-bromo-4-chloro-3-indolyl phosphate (BCIP, 0.4 mM). All sections were counterstained with 1% methyl green.

### Immunohistochemistry

Immunohistochemistry was performed as previously described^28^. The paraffin sections (5 μm) were deparaffinized in xylene, rehydrated with a graded series of ethanol, and washed in water. Antigen retrieval was performed by microwaving the sections in 10 mM sodium citrate buffer (pH 6.0) followed by cooling the sections to room temperature. The endogenous horse radish peroxidase activity was inhibited with 3% H_2_O_2_ for 15 min. After nonspecific binding was blocked with 10% horse serum at 37 °C for 1 h, the sections were incubated with a rabbit anti-p-Stat3 antibody (#9145, 1:400, Cell Signaling, USA) at 4 °C overnight. The sections were then incubated with a biotin-labeled goat anti-rabbit IgG antibody and a streptavidin-conjugated HRP complex (Zhongshan Golden Bridge, China). Finally, the signals were visualized with the DAB Horseradish Peroxidase Color Development Kit. The sections were counterstained with hematoxylin.

### Real-time PCR

The total RNA was isolated using the TRIzol Reagent Kit (Invitrogen), digested with RQ1 deoxyribonuclease I (Promega, Fitchburg, WI), and reverse-transcribed into cDNA with the PrimeScript Reverse Transcriptase Reagent Kit (TaKaRa, Japan). For real-time PCR, the cDNA was amplified using a SYBR Premix Ex Taq Kit (TaKaRa) on the CFX96 Touch™ Real-Time System (Bio-Rad). The data from real-time PCR were analyzed using the 2^−ΔΔCt^ method. The relative expression levels were normalized to the expression level of *Rpl7* (mouse) or *RPL7* (human).

### Western blot

Western blot was performed as previously described^21,22^. Briefly, the tissues or cells were lysed in lysis buffer (150 mM NaCl; 50 mM Tris-HCl, pH 7.5; 1% Triton X-100; and 0.25% sodium deoxycholate). The protein concentrations were measured with the BCA Kit (Thermo Fisher). The protein samples were separated on 10% SDS-PAGE gels and were transferred onto PVDF membranes. The membranes were incubated with primary antibody overnight at 4 °C. The primary antibodies used in this study include anti-phospho-Stat3 (#9145, 1:1000, Cell Signaling), anti-p53 (#2524, 1:1000, Cell Signaling), anti-Cytokeratin18 (#6259, 1:1000, Santa Cruz, USA), anti-Vimentin (#3932, 1:1000, Cell Signaling), anti-Tubulin (#2144, 1:1000, Cell Signaling), anti-β-Actin (#4970, 1:1000, Cell Signaling) and anti-GAPDH (#25778, 1:2000, Santa Cruz). After the membranes were incubated with an HRP-conjugated secondary antibody (1:5000) for 1 h, the signals were detected with an ECL Chemiluminescent Kit (Millipore, USA).

### Immunofluorescence

Immunofluorescence was performed as previously described with some modifications^21,22^. After the paraffin sections (5 μm) were deparaffinized and rehydrated, antigen retrieval was performed by microwaving the sections in 10 mM sodium citrate buffer (pH 6.0). Nonspecific binding was blocked with 3% BSA. The sections were incubated with a rabbit anti-NPM1 antibody (#10306, Proteintech, USA) in blocking solution overnight at 4 °C; then, the sections were incubated with an FITC-conjugated secondary antibody for 40 min. Finally, the sections were counterstained with 4′6-diamidino-2-phenylindole dihydrochloride (DAPI) or propidium iodide (PI) and were mounted with ProLong™ Diamond Anti-fade Mountant (Thermo Fisher, USA). The pictures were captured by laser scanning confocal microscopy (Leica, Germany).

### Lactate assay

The blastocysts were collected from uteri of pregnancy mice on day 4 and were cultured in the 25 μl 2% FBS culture medium, each drop contains 20 embryos. After 48 h, the lactate concentration of medium was assayed by L-Lactate Assay Kit (Cayman, USA) according to the manufacturer’s instructions. The assay was detected using a fluorescence spectrophotometer at excitation wavelength 530–540 nm and emission wavelength 585–595 nm.

### Statistical analysis

All of the experiments were repeated independently at least three times. For mouse studies, at least three mice were included in each group. The data were presented as the mean ± standard deviation (SD). The differences between the two groups were compared by Student’s *t*-test. A *P* value < 0.05 was considered statistically significant.

## Results

### ActD activation of delayed implantation via nucleolar stress

**Fig. 1:**
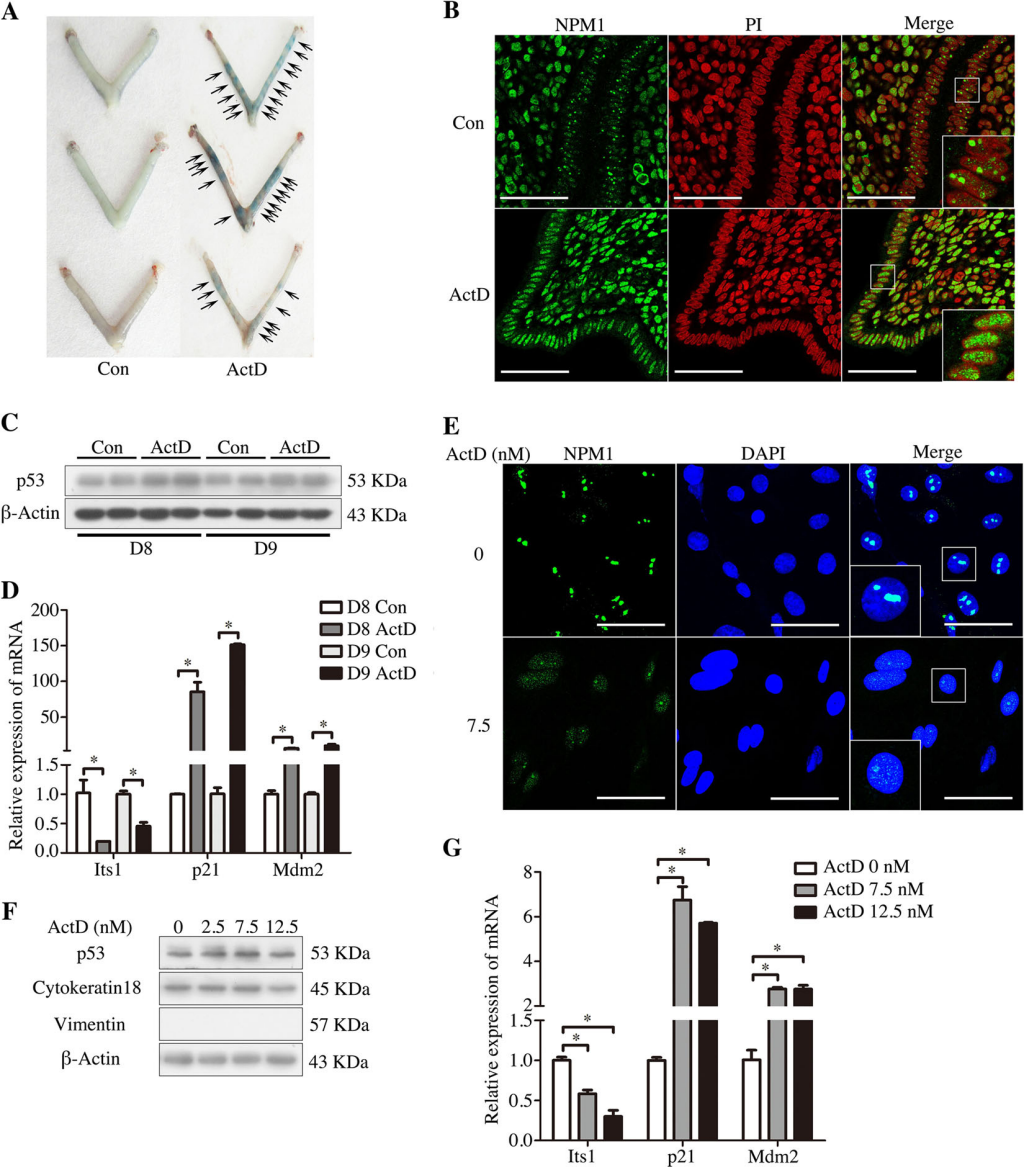
The effects of ActD-induced nucleolar stress on embryo implantation. **a** Delayed embryo implantation is activated by ActD at a dosage of 27.5 μg/mouse. **b** Immunofluorescence was used to indicate the location of NPM1 in the control and ActD-treated mouse uterus. Bar = 50 μm. **c** Western blot of the p53 protein in mouse uteri on days 8 and 9 (D8, mouse treated with vehicle or ActD for 1 day; D9, mouse treated with vehicle or ActD for 2 days). β-Actin was used as a loading control. **d** Real-time PCR analysis of the Its1, p21 and Mdm2 mRNA levels in mouse uteri on days 8 and 9. **e** The location of NPM1 in the luminal epithelial cells treated with ActD for 12 h. Bar = 25 μm. **f** Western blot of the p53 protein in ActD-treated luminal epithelial cells. Cytokeratin18 was used as a marker of luminal epithelium. Vimentin was used as a marker of stromal cells. β-Actin was used as a loading control. **g** Real-time PCR analysis of the Its1, p21 and Mdm2 mRNA levels in the ActD-treated luminal epithelial cells. Data are presented as the mean ± SD, *p < 0.05

Previous studies showed that the delayed implantation of mice and rats could be activated by ActD^18,19^. ActD is a selective inhibitor of polymerase I transcription and an inducer of nucleolar stress^6^. Therefore, we assumed that nucleolar stress may be involved during embryo implantation. To explore whether delayed implantation was activated by ActD, the mice with delayed implantation were treated with ActD on day 7. Compared to those of the control group, implantation sites were clearly observed in the ActD-treated group (Fig. 1a). In ActD-treated mice, NPM1, a marker of nucleolar stress, was relocated from the nucleolus to the nucleoplasm in the endometrial luminal epithelial cells on days 8 and 9 (Fig. 1b). Western blot analyses showed that p53 was upregulated in the ActD-treated uteri (Fig. 1c). Additional markers of nucleolar stress were also noted in these samples^17^. In the ActD-treated uteri, pre-rRNA (Its1) was downregulated, while p21 and Mdm2, the p53 target genes, were upregulated (Fig. 1d). These results suggested that nucleolar stress takes place in the ActD-treated uteri. When cultured luminal epithelial cells were treated with 2.5, 7.5, and 12.5 nM ActD, NPM1 was relocated from the nucleolus to the nucleoplasm after ActD treatment for 12 h (Fig. 1e). In these ActD-treated cells, there were an increase in the levels of p53, p21 and Mdm2 (Fig. 1f, g) and a decrease in Its1 (Fig. 1g). Overall, these data indicated that ActD could induce nucleolar stress in luminal epithelial cells.

### Effects of nucleolar stress on the Lif-STAT3 pathway

**Fig. 2:**
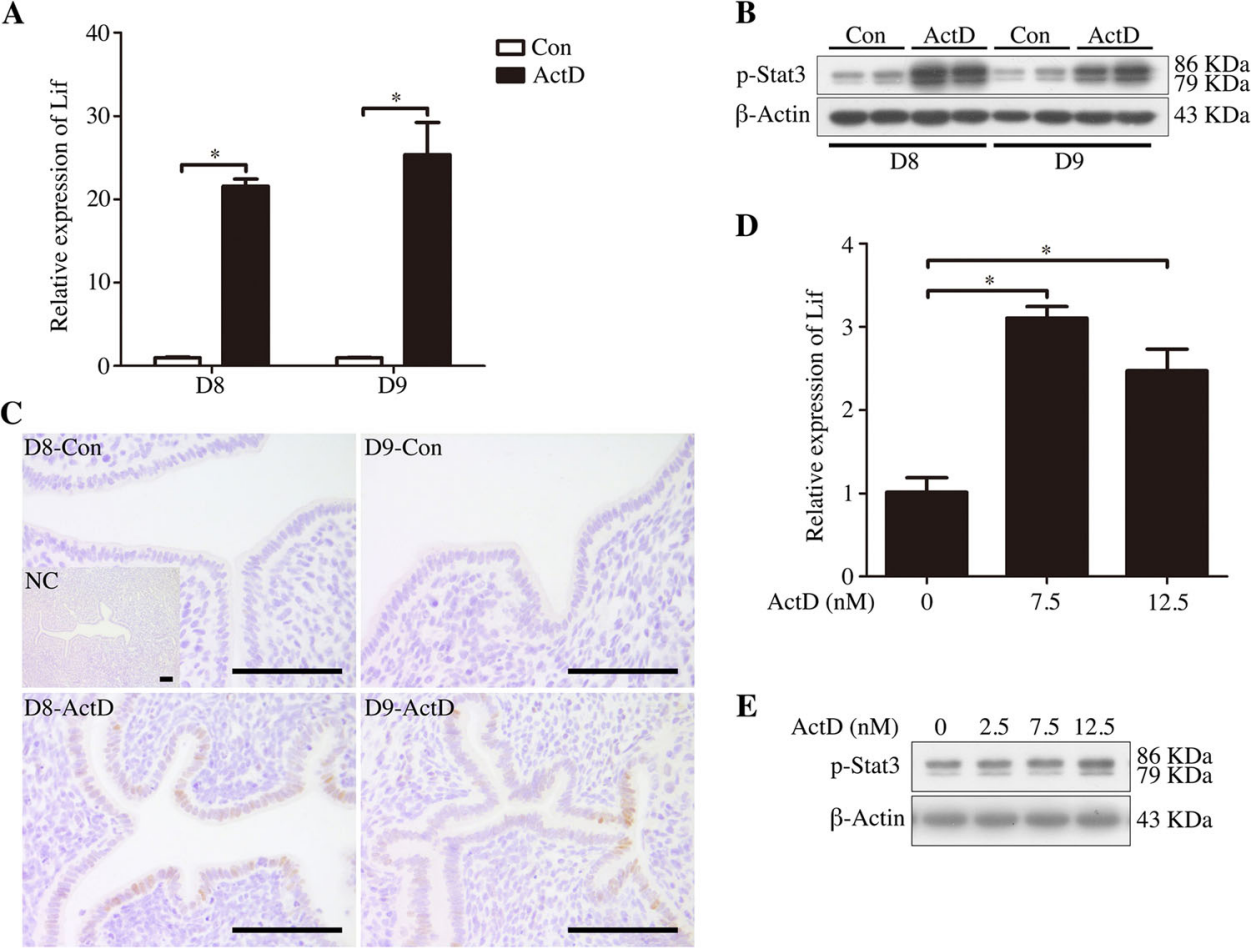
Effects of nucleolar stress on Lif and p-Stat3 levels after delayed implantation were activated by ActD. **a** Real-time PCR analysis of the Lif mRNA level in mouse uteri on days 8 and 9 (D8, mouse treated with vehicle or ActD for 1 day; D9, mouse treated with vehicle or ActD for 2 days). **b** Western blot of the p-Stat3 protein in mouse uteri on days 8 and 9. β-Actin was used as a loading control. **c** Immunohistochemical staining showing p-Stat3 immunostaining in mouse uteri on days 8 and 9. Bar = 100 μm. **d** Real-time PCR analysis of the Lif mRNA level in the luminal epithelial cells treated with ActD for 12 h. **e** Western blot of the p-Stat3 protein in luminal epithelial cells treated with ActD for 1 h. β-Actin was used as a loading control. Data are presented as the mean ± SD, *p < 0.05

Lif and its downstream target Stat3 are required for mouse embryo implantation^13,14^. When the mice under delayed implantation were treated with ActD on day 7, Lif expression was significantly increased in these ActD-treated uteri on days 8 and 9 (Fig. 2a). In these ActD-treated mouse uteri, the p-Stat3 level was also upregulated (Fig. 2b). Immunostaining also indicated that there was a significant increase in p-Stat3 nuclear localization in the luminal epithelium of the ActD-treated mice on days 8 and 9 (Fig. 2c). When the cultured luminal epithelial cells were treated with different concentrations of ActD, the levels of both Lif mRNA and p-Stat3 protein were significantly increased (Fig. 2d, e). These results suggest that the Lif-STAT3 signaling pathway should be activated by ActD treatment in the mouse uterus.

### Effects of ActD-soaked beads on embryo implantation

**Fig. 3:**
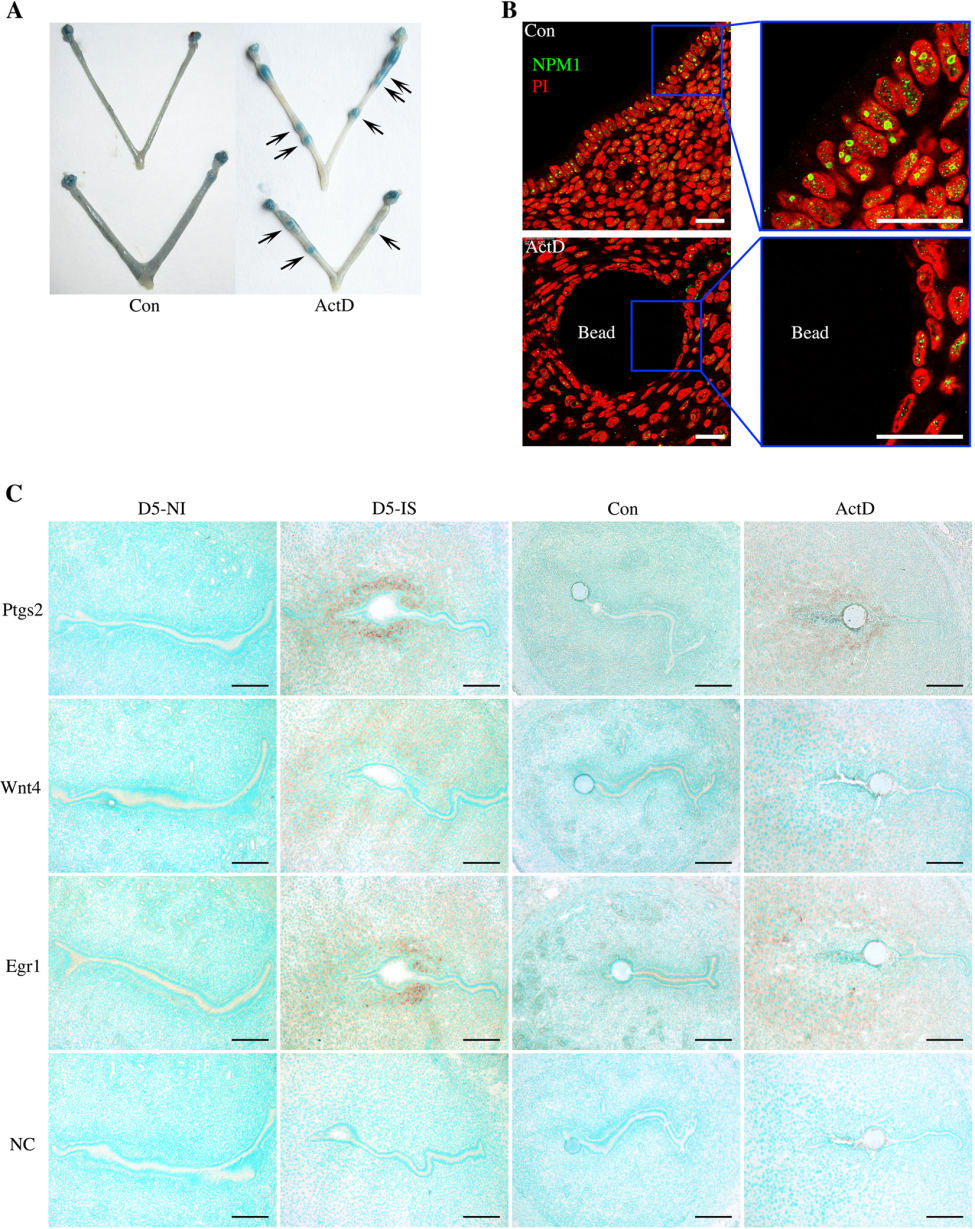
The effects of ActD-soaked beads on the attachment reaction in the mouse uterus. **a** The blue bands in the mouse uteri on day 6 after ActD (1 mM)-soaked beads were transplanted into the uteri of pseudopregnant mice on day 4. **b** NPM1 immunofluorescence after the transfer of ActD-soaked beads (right image, enlarged from the blue quadrate in the left image). Bar = 25 μm. **c** In situ hybridization of the Ptgs2, Wnt4 and Egr1 mRNA after the transfer of the ActD-soaked beads and day 5 of pregnancy. Bar = 100 μm

Because we showed that delayed implantation was activated by ActD, ActD-soaked beads were used to analyze the effects of nucleolar stress on embryo implantation. When ActD-soaked beads (1 mM) were transferred into the uterine lumen on day 4 of pseudopregnancy, these beads were very effective in inducing a blue reaction (blue bands) on day 6 (Fig. 3a). Compared to the effects in the control mice, the treatment of mice with ActD-soaked beads caused a translocation of NPM1 from the nucleolus to the nucleoplasm in the luminal epithelial cells around the beads (Fig. 3b). In ActD-treated uteri, there was an obvious induction of Ptgs2, Wnt4 and Egr1 mRNA expression in the stroma surrounding the beads, which expression patterns were similar to the implantation sites of day 5 of pregnancy (Fig. 3c). These results suggest that nucleolar stress could induce an implantation-like reaction and an implantation-related gene expression pattern.

### Nucleolar stress during natural embryo implantation

**Fig. 4:**
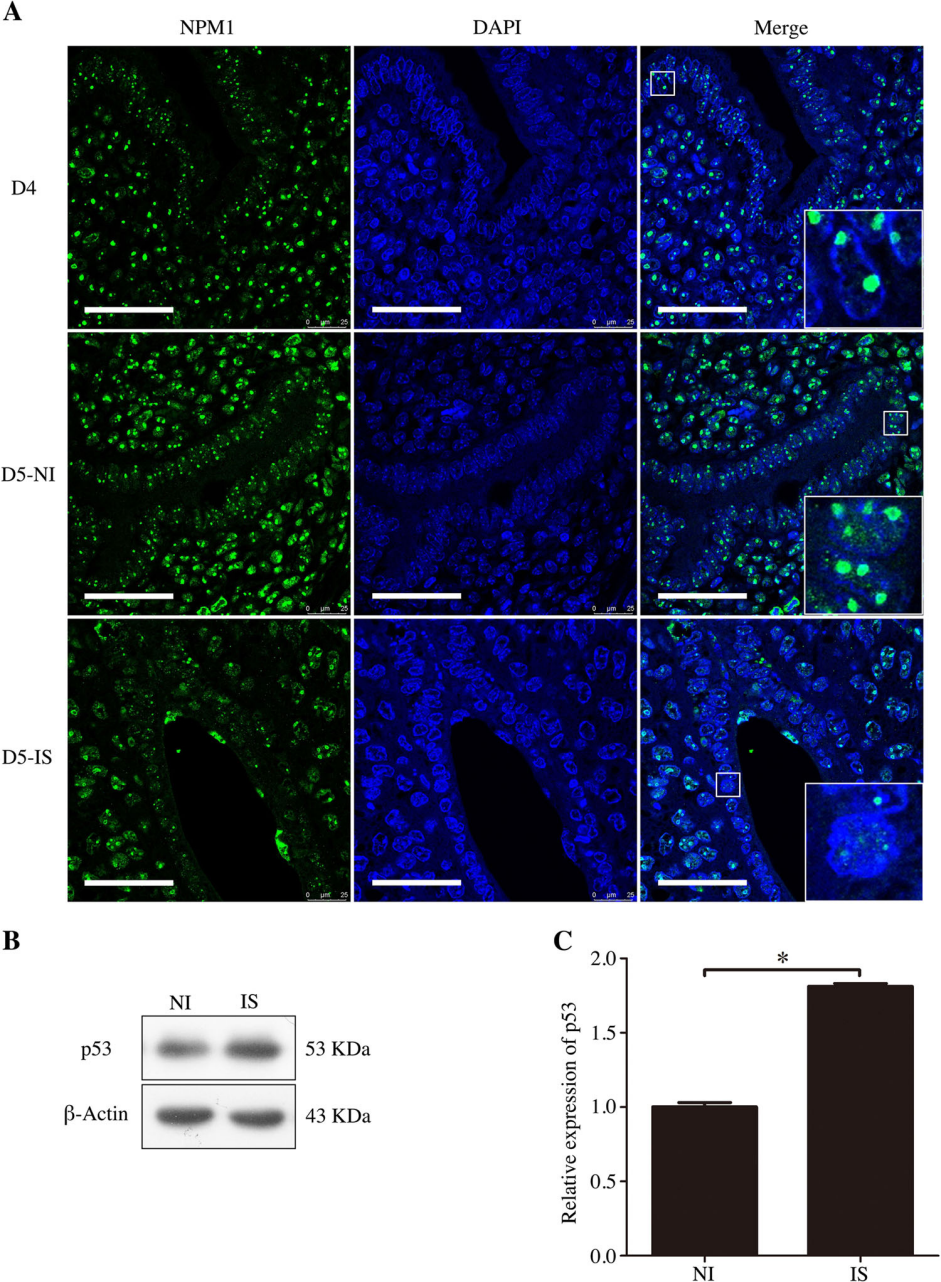
Nucleolar stress in the mouse uterus during the peri-implantation period. **a** Immunofluorescence was used to indicate the location of NPM1 in mouse uteri from days 4–5 of pregnancy (D5-NI, inter-implantation site on day 5; D5-IS, implantation site on day 5). Bar = 50 μm. **b** Western blot of the p53 protein in mouse uteri on day 5 of pregnancy. β-Actin was used as a loading control. **c** Real-time PCR analysis of the p53 mRNA level in the mouse uterus on day 5 of pregnancy. Data are presented as the mean ± SD, *p < 0.05

Because our data suggested that nucleolar stress is closely related to embryo implantation, we aimed to explore whether nucleolar stress was present during natural embryo implantation. On day 4 of pregnancy, NPM1 signals were mainly located in the nucleoli of endometrial cells. On day 5 of pregnancy, NPM1 signals were significantly decreased in the nucleoli of the luminal epithelium at the implantation sites, while NPM1 signals were also observed in the nucleoli at the inter-implantation sites (Fig. 4a). Compared to those of the inter-implantation sites, the protein and mRNA expression levels of p53 were obviously increased at the implantation sites on day 5 (Fig. 4b, c). These results suggest that nucleolar stress should exist during embryo implantation.

### Blastocyst-derived lactate induces nucleolar stress in luminal epithelial cells

**Fig. 5:**
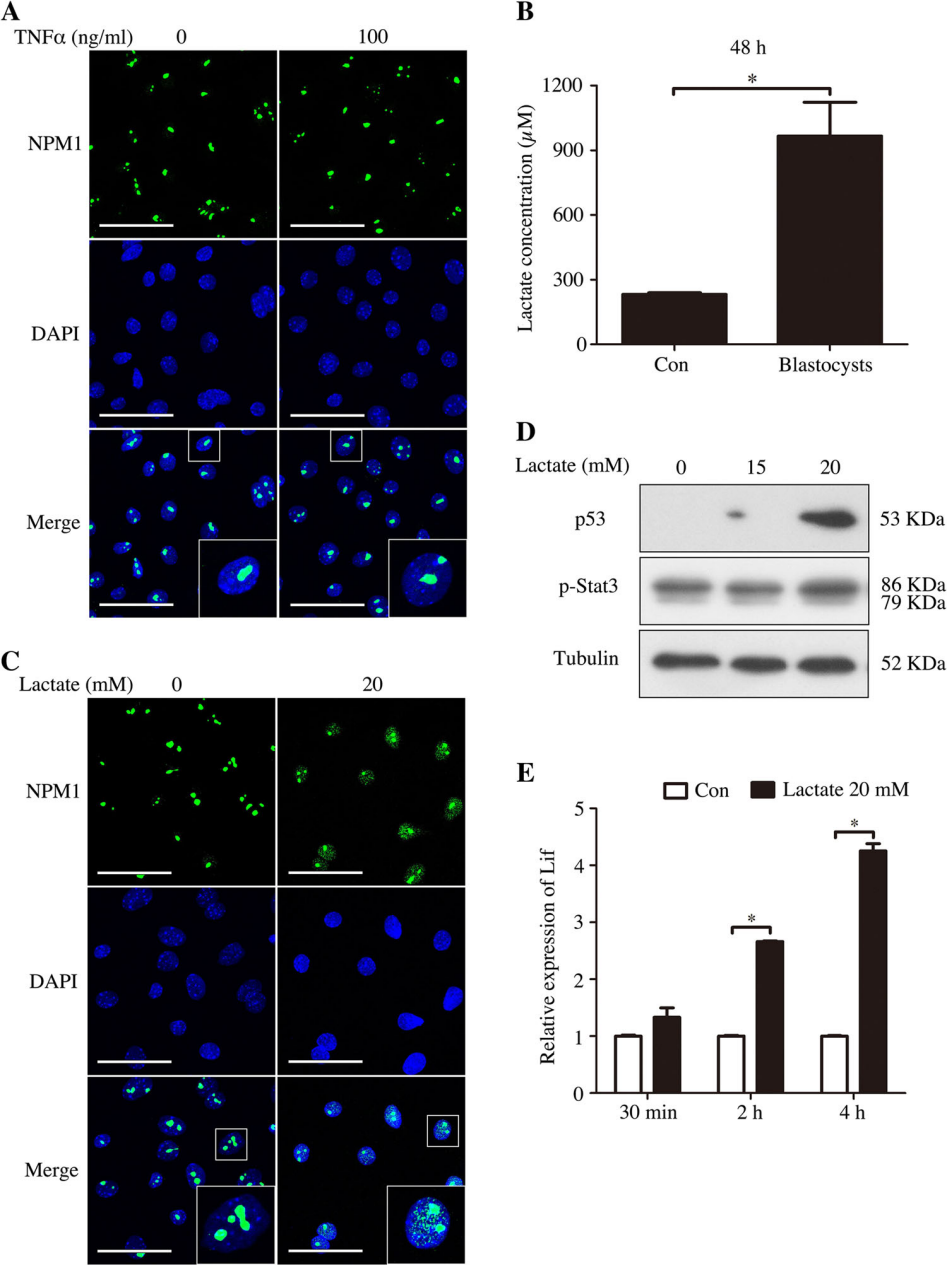
Induction of nucleolar stress in uterine luminal epithelial cells. **a** The location of NPM1 in the luminal epithelial cells treated with 100 ng/ml TNFα for 6 h. Bar = 25 μm. **b** The lactate concentration changes of medium after embryos cultured for 48 h. **c** The location of NPM1 in the luminal epithelial cells treated with 20 mM lactate for 2 h. Bar = 25 μm. **d** Western blot of the p53 and p-Stat3 proteins in the lactate-treated luminal epithelial cells. Tubulin was used as a loading control. **e** Real-time PCR analysis of the Lif mRNA level in the lactate-treated luminal epithelial cells. Data are presented as the mean ± SD, *p < 0.05

Our data showed that nucleolar stress exists during mouse embryo implantation. We wondered what initiates nucleolar stress during early pregnancy. TNFα is expressed and secreted from blastocysts^11,29^. However, TNFα treatment from 3 to 24 h had no effects on the translocation of NPM1 in the nucleolus (Fig. 5a) (the data of 3 h, 12 h, and 24 h were not shown). In mice, uterine acidification occurs during embryo implantation^30^. In human fertile endometrial fluid, Lactobacillus is the dominated microbiota^31^. Additionally, blastocysts can secrete lactate into the surrounding fluids during development^32^. Thus, we assumed that lactate may be involved in uterine acidification. In our study, the concentration of lactate in the culture medium was increased after the blastocysts of day 4 were cultured for 48 h (Fig. 5b). Therefore, we further analyzed the effects of lactate on nucleolar stress. When cultured epithelial cells were treated with 20 mM lactate for 2 h, NPM1 was partly relocated from the nucleolus to the nucleoplasm (Fig. 5c). Meanwhile, lactate treatment induced a significant increase in p53 and p-Stat3 (Fig. 5d). The expression of Lif mRNA was also upregulated by lactate treatment from 30 min to 4 h (Fig. 5e). These data suggested that nucleolar stress could be activated by lactate in the luminal epithelial cells.

### The expression and function of NPM1 in the peri-implantation uterus

**Fig. 6:**
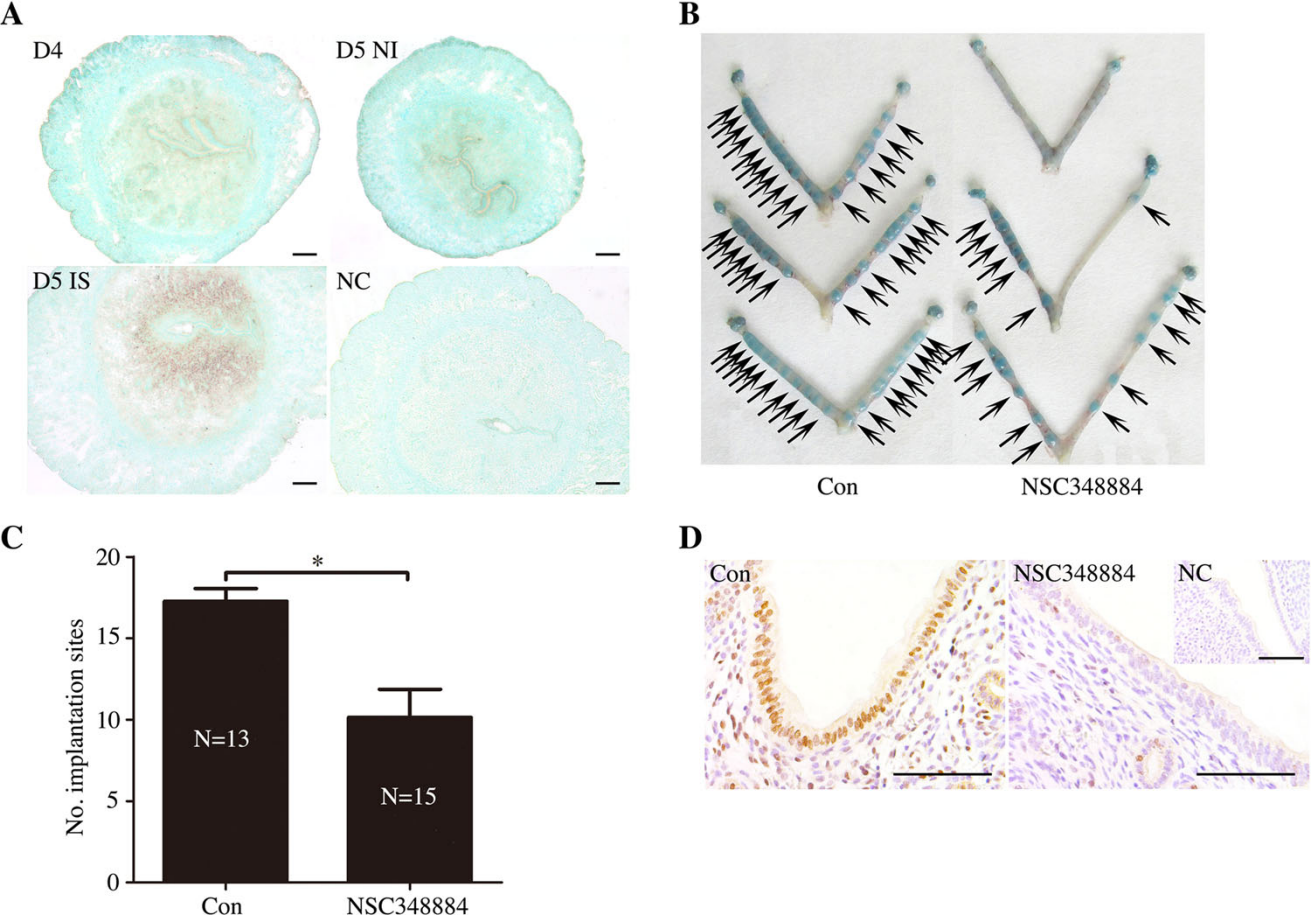
Npm1 expression and function during embryo implantation. **a** In situ hybridization on the mRNA localization of NPM1 in mouse uteri from days 4–5 of pregnancy. Bar = 100 μm. **b** The morphology of uteri on day 6 after the mice were treated with NSC348884 (160 μg/mouse) on days 4 and 5. **c** The mean number of implantation sites on day 6 under NSC348884 treatment (N, the number of mice). **d** Immunohistochemical staining was used to show the p-Stat3 protein in uteri on day 4 after the mice were treated with NSC348884 (160 μg/mouse) on day 3. Bar = 100 μm. Data are presented as the mean ± SD, *p < 0.05

The translocation of NPM1 in the nucleolus is a marker of nucleolar stress^11,17^. We would like to examine Npm1 expression in the mouse uterus. In situ hybridization showed that there was a basal level of Npm1 mRNA expression in the uterine endometrium on pregnancy day 4 and in the inter-implantation sites on pregnancy day 5. Npm1 was strongly expressed in the decidual cells at the implantation sites on pregnancy day 5 (Fig. 6a).

NSC348884 is a specific inhibitor of NPM1 that disrupts NPM1 oligomer formation^33^. When pregnant mice were treated with NSC348884 from days 4 to 5 of pregnancy, the number of implantation sites was significantly reduced (Fig. 6b, c). Immunostaining experiments showed that the level of p-Stat3 nuclear localization in the luminal epithelial cells on day 4 was significantly decreased in NSC348884-treated mice (Fig. 6d). These results show that NPM1 is important for mouse embryo implantation.

### Nucleolar stress could be induced inAN3 CA cells by low-dose ActD

**Fig. 7:**
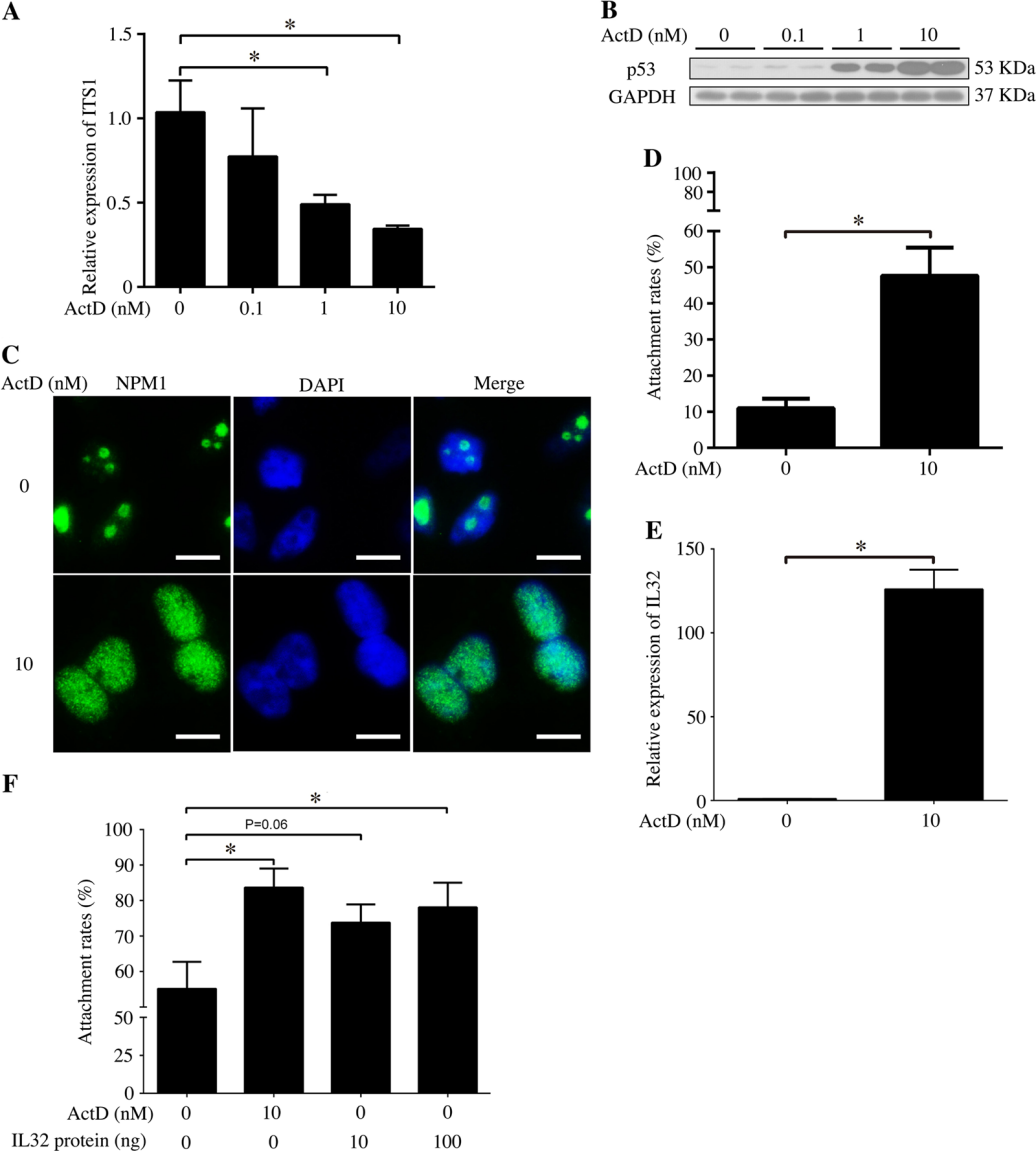
Induction of nucleolar stress in AN3 CA cells and effects of IL32 on trophoblast spheroid attachment to monolayer AN3 CA cells in vitro. **a** Real-time PCR analysis of the ITS1 mRNA level in the ActD-treated AN3 CA cells. **b** Western blot of the p53 protein in ActD-treated AN3 CA cells. GAPDH was used as a loading control. **c** The location of NPM1 in the AN3 CA cells treated with ActD for 48 h. Bar = 10 μm. **d** The rates of attached Jeg-3 spheroids to monolayer AN3 CA cells which are treated with 10 nM ActD for 48 h. **e** Real-time PCR analysis of the IL32 mRNA level in the ActD-treated AN3 CA cells. **f** The Jeg-3 spheroid attachment rates to AN3 CA treated with 10 nM ActD or different concentration of IL32 for 48 h. Data are presented as the mean ± SD, *p < 0.05

To translate our mouse data into humans, we examined nucleolar stress in human endometrium. AN3 CA cells, a non-receptive human endometrium epithelial cell line, were treated by a low dose of ActD in the concentration of 0.1, 1, and 10 nM for 48 h, respectively. Results showed that ActD could inhibit pre-rRNA (ITS1) transcription (Fig. 7a) and promote p53 protein accumulation at the 10 nM (Fig. 7b). Furthermore, 10 nM ActD could give rise to dislocation of NPM1 protein from the nucleolar to the nucleoplasm (Fig. 7c). All of results above suggested that low-dose ActD did induce a significant nucleolar stress in AN3 CA.

### Effects of nucleolar stress on adhesivity of AN3 CA cells to trophoblast spheroids

Embryo implantation can be induced by low-dose ActD in rats and mice^18,19^. Since nucleolar stress could be induced by low-dose ActD in human endometrium epithelial cell AN3 CA, it is very interesting to speculate whether nucleolar stress can promote human implantation in an in vitro embryo implantation model using AN3 CA and Jeg-3 spheroids co-culture system. Therefore, in present study, AN3 CA cells were treated with 10 nM ActD for 48 h in which concentration is confirmed to elicit nucleolar stress in AN3 CA cells. The results suggested that compared to control, Jeg-3 spheroids attachment rates to AN3 CA monolayer were significantly upregulated by ActD treatment (Fig. 7d).

### IL32 mediates the adhesion promotion effect of nucleolar stress on AN3 CA cell monolayer

In order to explore the mechanism by which ActD upregulates the Jeg-3 cell spheroids adhesion rates to AN3 CA monolayer, RNA-seq was performed in AN3 CA cells treated with ActD. The top 50 upregulated genes were listed, including IL32 (Table 2). IL32, a secretory adhesion molecular that could interact with the extracellular part of αVβ3 through RGD peptides^34^, was upregulated dramatically by ActD compared to control. The RNA-seq data were confirmed by real-time PCR. ActD could upregulate IL32 expression by more than 100 folds in AN3 CA cells (Fig. 7e). Based on the above results, it seems that it is IL32 that mediates the adhesion promoting effects of ActD. To ascertain the action of IL32, IL32 recombinant protein was introduced to analyze the effects of IL32 on the Jeg-3 spheroids attachment rates to AN3 CA cell monolayer. When AN3 CA cells were treated with 10 nM ActD, 10 nM, or 100 nM IL32, the attachment rate was significantly upregulated by 100 nM IL32 compared with control, in common with 10 nM ActD (Fig. 7f).

**Table 2:** The RNA-seq data

Serial no.	Symbol	Log2 ratio(Act D/DMSO)
1	GAGE5	13.63662462
2	NEDD8-MDP1	10.51569984
3	LOC554249	9.625708843
4	RPL17-C18orf32	9.027905997
5	FAM90A27P	8.864186145
6	LOC100506860	8.707359132
7	LOC541473	8.535275377
8	ATP6V1G2-DDX39B	8.535275377
9	GOLGA6L3	8.28077077
10	LOC100287834	7.95419631
11	HCST	7.845490051
12	ARL2-SNX15	7.665335917
13	FABP5	7.426264755
14	TMEM31	7.330916878
15	RPL3L	7.276124405
16	SYNJ2BP-COX16	7.257387843
17	LINC00854	7.21916852
18	ACP5	7.044394119
19	LIMS4	6.918863237
20	NPTX2	6.87036472
21	GALR2	6.804131021
22	LOC79999	6.741466986
23	PDLIM1	6.614709844
24	CDRT4	6.475733431
25	FRG2	6.475733431
26	HSD17B6	6.375039431
27	CLDN7	6.266786541
28	NHLH1	6.169925001
29	SENP3-EIF4A1	6.14974712
30	GIPC3	6
31	MPZ	5.977279923
32	RNU6-1	5.94641896
33	HCN4	5.930737338
34	MYBPC3	5.930737338
35	STX16-NPEPL1	5.906890596
36	ST20-MTHFS	5.700439718
37	ZNF559-ZNF177	5.672425342
38	FGR	5.672425342
39	GGT8P	5.339850003
40	MUC22	5.247927513
41	ZNF582	5.087462841
42	LMTK3	5.087462841
43	IL32	5.069673528
44	ELAVL3	5.044394119
45	LOC286059	4.977279923
46	CEND1	4.727584358
47	PDGFRA	4.700439718
48	CDC42BPG	4.64385619
49	COL1A1	4.64385619
50	PLK2	4.558368291

### The effects of nucleolar stress on human endometrium epithelial cells with unequal receptivity

**Fig. 8:**
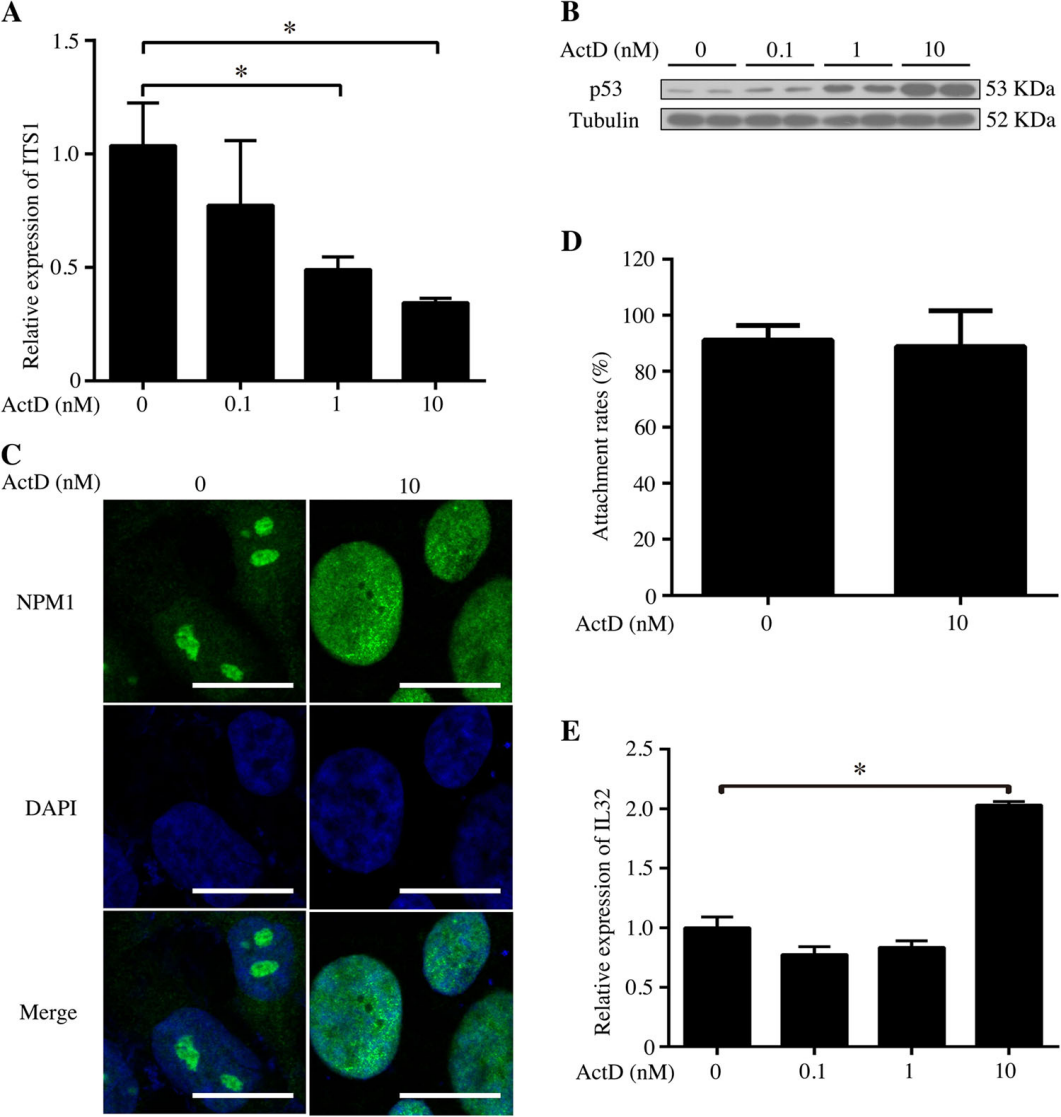
The effects of nucleolar stress on the human Ishikawa epithelial cells. **a** Real-time PCR analysis of ITS1 mRNA level in the ActD-treated Ishikawa cells. **b** Western blot of the p53 protein in ActD-treated Ishikawa cells. Tubulin was used as a loading control. **c** The location of NPM1 in the Ishikawa cells treated with ActD for 48 h. Bar = 10 μm. **d** The rates of attached Jeg-3 spheroids to monolayer Ishikawa cells which are treated with 10 nM ActD for 48 h. **e** Real-time PCR analysis of the IL32 mRNA level in the ActD-treated Ishikawa cells. Data are presented as the mean ± SD, *p < 0.05

Our data showed that nucleolar stress is beneficial for the adhesion of AN3 CA. Because AN3 CA cell is a non-receptive cell line, we explored whether data from AN3 CA cells could be applied to receptive cell lines. Ishikawa cell line is a well-differentiated steroid-responsive endometrial cell line with characteristics of luminal epithelium and glandular epithelium which represent a receptive endometrium^35^. When Ishikawa cells were treated with ActD for 48 h, ITS1 transcription was reduced (Fig. 8a), followed by an increase of p53 protein (Fig. 8b) and NPM1 translocation from the nucleolus to the nucleoplasm (Fig. 8c). However, ActD-induced nucleolar stress had no effects on the adhesivity of Ishikawa monolayer to trophoblast spheroids (Fig. 8d). Because IL32 was shown to mediate the adhesive effect of nucleolar stress on AN3 CA, we checked the level of IL32 expression in Ishikawa cell line. After Ishikawa cells were treated with ActD, IL32 expression increased, but only around 2-folds in Ishikawa cells by nucleolar stress (Fig. 8e).

### The NPM1 expression in human endometrium during menstrual cycle

Because NPM1 is a multi-functional chaperone in nucleolus and plays crucial roles in nucleolar stress response^11^. We examined NPM1 expression pattern in human endometrium. Results demonstrated that NPM1 protein expression was detected in human endometrium during the menstrual cycle, showing a gradual increasing manner from proliferation phase to late secretory phase, especially highly expressed in luminal epithelium (Supplemental Fig. S1).

## Discussion

Previous studies have demonstrated that the delayed implantation of mice and rats can be activated by ActD^18,19^. However, the underlying mechanism was unclear. In this study, we show that delayed implantation can be activated by ActD-induced nucleolar stress. To our knowledge, this should be the first study to demonstrate that nucleolar stress plays a key role during embryo implantation.

NPM1 is located in the nucleoli of cells under normal conditions. During nucleolar stress, NPM1 relocates from the nucleolus to the nucleoplasm^11,17^. In the nucleoplasm, NPM1 can combine with Mdm2 and prevent it from degrading p53, resulting in p53 accumulation^8^. Therefore, the relocation of NPM1 from the nucleolus to the nucleoplasm and p53 accumulation are two hallmark events of nucleolar stress^11,17^. In our study, both NPM1 translocation and p53 accumulation were detected in the luminal epithelium at implantation sites on day 5 of pregnancy and during the activation of delayed implantation. We also found that both Lif mRNA and p-Stat3 protein levels were upregulated by ActD. In mice, p53 (the upstream of the Lif-STAT3 signaling pathway) is essential for the establishment of uterine receptivity^14,16^. p53 deficiency in C57 background mice results in a reduction in embryo implantation and pregnancy rate, which can be rescued by the administration of LIF^16^. Maternal Lif is strongly expressed in the glandular epithelium and is required for mouse embryo implantation^13^. The conditional knockout of uterine Stat3 also causes an embryo implantation failure^14,15^. In our study, ActD-soaked beads also initiated an implantation reaction in the mouse uterus and upregulated the levels of both Lif mRNA and p-Stat3 protein. To our knowledge, our data are the first to demonstrate that ActD-induced nucleolar stress is present during early pregnancy and plays a key role in embryo implantation.

The dialog between the blastocyst and the receptive uterus is essential for successful implantation^1^. Lactate is a main product of glycolysis^36,37^. Monocarboxylate transporters (Mct) facilitate the transport of lactate between the extracellular and intracellular environments^32^. Mct4, a lactate exporter, is expressed in the mouse blastocyst^38^. The blastocyst can secrete lactate into the surrounding fluids during development. Lactate may be related to uterine acidification during embryo implantation^32^. Our data also indicate that lactate has a time-dependent induction of Lif expression in luminal epithelial cells. Additionally, the nucleolar stress in the luminal epithelial cells is induced by lactate. Therefore, our data suggest that blastocyst-secreted lactate may induce nucleolar stress in the luminal epithelial cells, which should be important for embryo implantation.

NPM1 is a nucleolar phosphoprotein that localizes in the granular regions of the nucleolus^39^. The function of NPM1 requires dimer and pentamer formation by oligomerization^33^. NPM1 oligomerization can effectively activate STAT3^40^. In our study, Npm1 is strongly expressed in the primary decidua at implantation sites. When pregnant mice are treated with NSC348884, a specific inhibitor of NPM1^33^, the number of implantation sites is obviously inhibited. The level of p-Stat3 nuclear localization in the uterine luminal epithelium on day 4 of early pregnancy is also suppressed by NSC348884. Therefore, our data imply that NPM1 may play a key role during embryo implantation via nucleolar stress.

Although human embryo implantation cannot be studied in vivo due to ethical limitation, accumulating evidences validate the reliability and reproducibility of in vitro co-culture model of embryo-endometrial attachment in previously published studies^41,42^. The present study demonstrated that in vitro treatment with ActD has a significant promoting effect on embryo-endometrial attachment in a Jeg-3 spherods-AN3 CA cell co-culture model. However, this adhesion-promoting effect is not demonstrated within Jeg-3 spheroids-Ishikawa co-culture system. These results suggested that high-receptive epithelial cells (Ishikawa) are less sensitive than low receptivity epithelial cells (AN3 CA) to nucleolar stress induced by low dose of ActD. The nucleolar stress activated by canonical inducer, such as low dose of ActD, may have a beneficial effect on patients with poor endometrium receptivity which is the leading cause of female infertility, including recurrent implantation failure^43,44^. Furthermore, the increasing evidence demonstrates that an improvement in implantation rates or clinical pregnancy rate was showed in patients with a history of recurrent pregnancy loss or unexplained miscarriage who received endometrial scratch injury^45,46^. A similarity between nucleolar stress and endometrial scratch injury is that they are both effective only in patients with poor endometrium receptivity. Accordingly, it is postulated that there may have some inner relations between endometrial scratch injury and nucleolar stress response which is worthy of further investigation. Notwithstanding, the data gained from in vitro cell culture models could not be directly introduced to human beings. In view of unavailability of actual human blastocysts for such experimental use due to ethical reason, the present study may, more or less, shed a light on the remedy of recurrent implantation failure patients with low endometrium receptivity.

In our study, data from transcriptomic sequencing indicated that there are 380 upregulated genes with at least 4 folds in ActD-treated group compared to control. Among the upregulated genes, IL32 is one of the top 50 upregulated genes (Table 2). IL32 is a proinflammatory cytokine that could be secreted^47^. Besides, IL32 contains an RGD motif which interacts with the extracellular domain of integrins including αVβ3 and αVβ6 integrins^34^. Therefore, IL32 is selected as a candidate that mediates the adhesion promoting effects of nucleolar stress on the co-culture system of Jeg-3 spheroids and AN3 CA cell monolayer. Increasing evidence demonstrates that IL32 has a positive effect on invasion and migration in breast cancer cells and human gastric cancer cells^48,49^.

There are plenty of immunology accommodation and inflammatory reaction existing during embryo implantation. Stress response may exist in the normal physiological process and play essential parts in embryo implantation if these stress responses are fine-tuned. We previously demonstrated that a proper endoplasmic reticulum stress is important for mouse embryo implantation^21^. In present study, nucleolar stress is induced successfully by ActD in Ishikawa and AN3 CA which are on behalf of high-receptive and low-receptive human endometrium epithelial cells, respectively. Nucleolar stress has a positive effect on embryo implantation in a widely used human in vitro implantation model. Moreover, the important nucleolar stress effector NPM1 is highly expressed in the mid- or late secretory human endometrium, especially in lumen epithelial cell nucleolus. These data suggested that physiological nucleolar stress response may play positive roles in human implantation process.

In summary, our data indicate that lactate-induced nucleolar stress may regulate mouse embryo implantation through the p53-Lif-STAT3 pathway. The localized NPM1 expression at implantation sites should be important for embryo implantation via mediating nucleolar stress. Furthermore, nucleolar stress may play a positive role in human embryo adhesion to endometrial epithelial cell monolayer through IL32.

## Supplementary information

Supplementl figure legends for Figure S1

Supplemental Figure S1
