# Supplementary material for: Nucleolar stress regulation of endometrial receptivity in mouse models and human cell lines
Source: Cell Death Dis. 2019 Nov 4;10(11):831. doi: 10.1038/s41419-019-2071-6 (PMC6828743; doi:10.1038/s41419-019-2071-6)
Supplement: Supplementary file 1 — Supplementl figure legends for Figure S1 [file 41419_2019_2071_MOESM1_ESM.docx]

**Supplemental Figure S1: NPM1 protein expression in human endometrium during menstrual cycle.** PP, proliferation phase; ESP, early secretory phase; MSP, middle secretory phase; LSP, late secretory phase. Bar = 30 μm.
